# Bayesian Analysis of Tweedie Compound Poisson Partial Linear Mixed Models with Nonignorable Missing Response and Covariates

**DOI:** 10.3390/e25030506

**Published:** 2023-03-15

**Authors:** Zhenhuan Wu, Xingde Duan, Wenzhuan Zhang

**Affiliations:** Department of Mathematics and Statistics, Guizhou University of Finance and Economics, Guiyang 550025, China

**Keywords:** random effects, Tweedie compound Poisson distribution, Bayesian P-spline, longitudinal semicontinuous data, logistic regression model

## Abstract

Under the Bayesian framework, this study proposes a Tweedie compound Poisson partial linear mixed model on the basis of Bayesian P-spline approximation to nonparametric function for longitudinal semicontinuous data in the presence of nonignorable missing covariates and responses. The logistic regression model is simultaneously used to specify the missing response and covariate mechanisms. A hybrid algorithm combining the Gibbs sampler and the Metropolis–Hastings algorithm is employed to produce the joint Bayesian estimates of unknown parameters and random effects as well as nonparametric function. Several simulation studies and a real example relating to the osteoarthritis initiative data are presented to illustrate the proposed methodologies.

## 1. Introduction

Semicontinuous data, characterized by nonnegative continuous value with a discrete mass of zero, appear frequently in many fields, such as medicine, health, economics, and ecology. Models for longitudinal semicontinuous data have, in particular, been receiving a lot of attention in two ways. The first approach is the two-part mixed model wherein a mixture of Bernoulli with positive support distribution is used to model zero and positive components separately (Olsen and Schafer [1]; Berk and Lachenbruch [2]; Tooze et al. [3]; Su et al. [4,5]; Liu et al. [6]; Zhou et al. [7]). However, Hasan et al. [8] and Yan and Ma [9] pointed out that such artificial separation based on the two-part modeling method breaks down the serial patterns in the analysis of time series and longitudinal data. The second approach is the compound Poisson mixed model for modelling longitudinal and repeated measurement or cluster data in an integral way. For example, Zhang [10] investigated several statistical inference methods for Tweedie compound Poisson linear mixed models from the frequentist and Bayesian perspective. Swallow et al. [11] developed a Bayesian hierarchical Tweedie regression model by incorporating serial temporal and spatial correlation into the Tweedie distribution in the analysis of longitudinal semicontinuous ecological data. Ye et al. [12] investigated the sensitivity analysis for priors in Tweedie compound Poisson random effect models under a Bayesian framework. In particular, Yan and Ma [9] incorporated serially dependent distribution-free random effects into the compound Poisson regression model for longitudinal semicontinuous data. However, all the abovementioned compound Poisson mixed models have limitations in that they either do not consider nonlinear smooth effects of covariates, such as time and age variables, or do not deal with missing responses and covariates.

It is well known that handling missing data has become an active research field in data analysis. Many methods have been proposed to make statistical inference on various regression models with nonignorable missing response or covariates. For example, Ibrahim et al. [13,14] proposed two methods by which to estimate unknown parameters in generalized linear models with nonignorable missing covariates and generalized linear mixed models with nonignorable missing responses by using the EM algorithm, respectively. In addition, based on these frequentist approaches of handling nonignorable missing response or covariate data, their Bayesian analogues have been extended to various regression models. For example, from a Bayesian perspective, see Huang et al. [15] for generalized linear models with nonignorably missing covariates, Lee and Tang [16] for nonlinear structural equation models with nonignorable missing data, Tang and Zhao [17] for nonlinear reproductive dispersion mixed models for longitudinal data with nonignorable missing covariates, Tang et al. [18] for a nonlinear dynamic factor analysis model with nonparametric prior and possible nonignorable missingness, Zhou et al. [7] for two-part hidden Markov models for semicontinuous longitudinal data with nonignorable missing covariates, Wang and Tang [19] for Bayesian quantile regression with mixed discrete and nonignorable missing covariates, and Wang et al. [20] for Bayesian latent factor on image regression with nonignorable missing data. Therefore, we propose a fully Bayesian method by which to simultaneously estimate unknown parameters, random effects and nonparametric function in a Tweedie compound Poisson partial linear mixed models on the basis of Bayesian P-spline approximation to nonparametric function in the presence of nonignorable missing covariates and responses, where the nonignorable missing data mechanism is specified by a logistic regression model.

For the sake of brevity and readability, we first introduce the main mathematical symbols and their descriptions in the rest of paper summarized in Table 1.

The paper is organized as follows. In Section 2, we give a description of the data. In Section 3, we describe a Tweedie compound Poisson partial linear mixed models in the presence of nonignorable missing covariates and responses. We present the Bayesian P-spline to model the nonparametric function. The logistic regression model is simultaneously used to specify the missing response and covariate mechanisms, and a sequence of one-dimensional conditional distributions is used to model the joint probability function of the missing covariates. In Section 4, the prior distributions and posterior distributions of unknown parameters and latent variables are presented. In Section 5, two simulation studies and an example are given to illustrate our proposed methodologies. In Section 6, we give some conclusions. In the Appendix A and Appendix B, the conditional distributions for Gibbs sampling and the Metropolis–Hastings algorithm are given.

## 2. Data Description

In this section, we describe the Osteoarthritis Initiative (OAI) database, which is available at https://www.oai.ucsf.edu (accessed on 4 April 2017). The OAI cohort study investigated the causes of knee osteoarthritis for 4796 patients aged 45 and older, and collected some information such as age, sex, and body mass index (BMI) for these patients at baseline, 12 months, 24 months, 36 months, and 48 months. Thus, this information is collected at most five times because of the missing data involved. In addition, this OAI study adopted the Western Ontario and McMaster Universities Arthritis Index (WOMAC) disability scores to assess the pain intensity in these patients with hip and/or knee osteoarthritis. Higher scores on the WOMAC score indicate worse pain, stiffness, and functional limitations for these patients. A sample of two patients (denoted by ID 9019406 and ID 9025191) from the OAI study is presented in Table 2.

**Figure 1:**
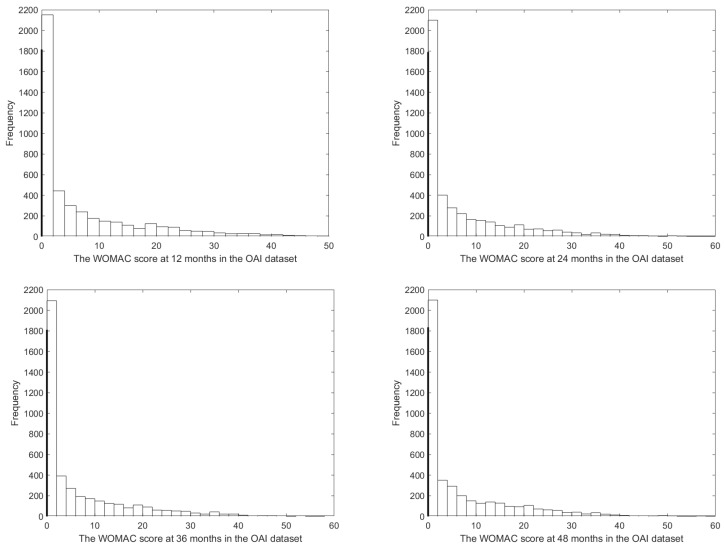
Histogram for the observed WOMAC numeric score in the OAI dataset.

The missing rates for the longitudinal WOMAC scores outcome at baseline, 12 months, 24 months, 36 months, 48 months are 0.3%, 7.1%, 10.9%, 12.1%, and 12.3%, respectively. Moreover, the missing rates for covariate BMI at five different time points are 0%, 11.5%, 16.0%, 18.9%, and 21%, respectively. It can be seen from Figure 1 that the observed WOMAC numeric score at 12 months, 24 months, 36 months, and 48 months are right-skewed with a large numerical proportion of zeros, where the bold line on the left of each histogram denotes the frequency for zero. Specifically, more than 36.9% of the observations of all time points are zeros; thus we consider the WOMAC numeric score as a longitudinal semicontinuous response with missing data in this article.

## 3. Statistical Models

### 3.1. Tweedie Compound Poisson Distribution

As in Ma and Jørgensen [21], the probability density function of the Tweedie compound Poisson distribution has the following form,

(1) $$f_{p}\left(y;\mu ,\phi \right)=c_{p}\left(y;\phi \right)\exp\left\{\frac{1}{\phi }\left(\frac{y\mu^{1-p}}{1-p}-\frac{\mu^{2-p}}{2-p}\right)\right\},$$

where *p* is the power parameter satisfying 1<p<2, μ and ϕ are the mean parameter and dispersion parameter, respectively, and the expression for $c_{p}\left(y;\phi \right)$ is not analytically tractable when y>0. If a nonnegative random variable *Y* is distributed as a Tweedie compound Poisson distribution, then we simply denote $Y∼{Tw}_{p}\left(\mu ,\phi \right)$ in the rest of paper. Moreover, we have E(Y)=μ and $\mathrm{Var}\left(Y\right)=\phi \mu^{p}$. Furthermore, the random number *Y* of the Tweedie compound Poisson distribution is readily generated from the following stochastic representation

(2) $$Y=\sum_{i=1}^{U}X_{i},$$

where *U* is distributed as a Poisson distribution with mean λ, $X_{i}$ is the independent and identically distributed gamma distribution with mean αγ and variance $\alpha \gamma^{2}$, and *U* and $X_{i}$ are assumed to be independent. After some calculations, the relationship between the two sets of parameters in Equations (1) and (2) are derived as

(3) $$\begin{array}{c} \mu =\lambda \alpha \gamma \;\;\lambda =\frac{\mu^{2-p}}{\phi (2-p)}\\ p=\frac{\alpha +2}{\alpha +1}\;\;\;\;\alpha =\frac{2-p}{p-1}\\ \phi =\frac{\lambda^{1-p}{\left(\alpha \gamma \right)}^{2-p}}{2-p}\;\;\;\;\gamma =\phi \left(p-1\right)\mu^{p-1}. \end{array}$$

It follows from Equation (2) that the joint probability distribution of $\left(Y,U\right)$ is given by

(4) $$\begin{array}{c} p_{Y,U}\left(y,u|\lambda ,\alpha ,\gamma \right)=p_{Y|U}\left(y|u,\alpha ,\gamma \right)\times p_{U}\left(u|\lambda \right)\\ =\left\{\begin{array}{cc} \exp(-\lambda ) & \left(0,0\right)\\ \frac{y^{u\alpha -1}\exp\left(-y/\gamma \right)}{\Gamma \left(u\alpha \right){\left(\gamma \right)}^{u\alpha }}\times \frac{\lambda^{u}}{u!}\exp\left(-\lambda \right) & R^{+}\times Z^{+}. \end{array}\right. \end{array}$$

Thus, the marginal distribution of $\left(Y,U\right)$ has the abovementioned form given in Equation (1).

### 3.2. The Model

For modeling, we first introduce some notations. Let $y_{ij}$ be the longitudinal semicontinuous outcome with missing data of the *i*th patient with osteoarthritis measured at time $t_{ij}$ ($i=1,\ldots ,n,j=1,\ldots ,n_{i}$). In the OAI study, n=4796 is the number of patients with $n_{i}=5$ denoting the number of repeated observations per patient. Given random effects $b_{i}$, $Y_{i1},\ldots ,Y_{in_{i}}$ are conditionally independent and each $Y_{ij}|b_{i}$ is assumed to be the Tweedie compound Poisson distribution, that is

(5) $$\begin{array}{ccc} Y_{ij}|b_{i} & ∼ & {\mathrm{Tw}}_{p}\left(\mu_{ij},\phi \right), \end{array}$$

where $\mu_{ij}$ is the conditional expectation of the response $Y_{ij}$, ϕ is the dispersion parameter to be estimated and 1<p<2. Inspired by GLMM method, the conditional expectation $\mu_{ij}$ is modeled by

(6) $$\log\left(\mu_{ij}\right)=\eta_{ij}=x_{ij}^{T}\beta +z_{ij}^{T}b_{i}+g\left(t_{ij}\right),$$

where β is a q×1 vector of unknown regression parameter of interest, $x_{ij}$ is a q×1 vector of covariates in the presence of missing data, $b_{i}$ is distributed as $N_{r}\left(0,\Sigma \right)$, $z_{ij}$ is a r×1 vector of covariates relating to the random effects $b_{i}$, and $g(t_{ij})$ denotes an unknown nonparametric function satisfying the twice-differentiable property in term of time effects $t_{ij}$. In this article, the model defined in Equations (1) and (2) is referred to as a Tweedie compound Poisson partial linear mixed model.

Inspired by Lang and Brezger [22], we used the Bayesian P-spline method based on a linear combination of B-spline basic functions to approximate the unknown nonparametric function, that is

(7) $$g\left(t_{ij}\right)=\sum_{h=1}^{H}\xi_{h}B_{h}\left(t_{ij}\right),$$

where $B_{h}\left(\cdot \right)$ is the *h*th B-spline basis function, *H* is the number of B-spline basis function, and $\xi_{h}$ is the B-spline coefficients to be estimated. Under the Bayesian framework, $\xi_{h}$ is treated as a random variable, and defined by the following first-order random walk; that is, $\xi_{h}=\xi_{h-1}+v_{h}$, where $v_{h}∼N\left(0,\tau_{\xi }^{2}\right)$ for h=2,…,H and the diffuse prior $\xi_{1}$ is proportional to constant. The variance parameter $\tau_{\xi }^{2}$ is viewed as a global smoothing parameter. Although it is easy to estimate the global smoothing parameter, this global smoothing parameter is difficult to characterize in terms of the highly oscillating features for the underlying nonparametric functions g(t). To overcome this issue, we introduce the additional hyperparameters $\delta_{h}$ as local smoothing parameters, which can improve the estimation of a function with significantly different curvatures at different points $t_{ij}$. Thus, $\upsilon_{h}$ is assumed to be the normal distribution with heterogeneous variance; that is, $\upsilon_{h}∼N\left(0,\tau_{\xi }^{2}/\delta_{h}\right)$ for h=2,…,H. Furthermore, let $\xi ={\left(\xi_{1},\ldots ,\xi_{H}\right)}^{T}$ and $\delta ={\left(\delta_{2},\ldots ,\delta_{H}\right)}^{T}$. The prior distribution for ξ is derived in the matrix form

$$\xi |\tau_{\xi }^{2}\propto \exp\left(-\frac{1}{2\tau_{\xi }^{2}}\xi^{T}Q\xi \right),$$

where the penalty matrix ***Q*** is given by

$$Q=\left(\begin{array}{cccccccc} \delta_{2} & -\delta_{2} & 0 & 0 & \cdots & 0 & 0 & 0\\ -\delta_{2} & \delta_{2}+\delta_{3} & -\delta_{3} & 0 & \cdots & 0 & 0 & 0\\ 0 & -\delta_{3} & \delta_{3}+\delta_{4} & -\delta_{4} & \cdots & 0 & 0 & 0\\ \vdots & \vdots & \vdots & \vdots & \vdots & \vdots & \vdots & \vdots \\ 0 & 0 & 0 & 0 & \cdots & -\delta_{H-2} & \delta_{H-2}+\delta_{H-1} & -\delta_{H-1}\\ 0 & 0 & 0 & 0 & \cdots & 0 & -\delta_{H-1} & \delta_{H-1}+\delta_{H}\\ 0 & 0 & 0 & 0 & \cdots & 0 & 0 & -\delta_{H} \end{array}\begin{array}{c} 0\\ 0\\ 0\\ \vdots \\ 0\\ -\delta_{H}\\ \delta_{H} \end{array}\right).$$

Here, the prior distribution of smooth parameter $\tau_{\xi }^{2}$ is distributed as an inverse gamma distribution; that is, $p\left(\tau_{\xi }^{2}\right)∼IG\left(a_{\tau },b_{\tau }\right)$.

### 3.3. Missing Data Mechanism Assumptions

In this article, let $y_{i}={\left(y_{i1},\ldots ,y_{in_{i}}\right)}^{T}$ be a $n_{i}\times 1$ vector of response (i=1,…,n), and $x_{ij}$ be a q×1 vector of covariates in the presence of missing data, respectively, whereas $z_{ij}$ are completely observed. In what follows, we assume that the missing data mechanism for response and covariates are nonignorable. Let $y_{i}={\left(y_{oi}^{T},y_{mi}^{T}\right)}^{T}$ and $x_{ij}={\left(x_{oij}^{T},x_{mij}^{T}\right)}^{T}$, where $y_{oi}\left(n_{1i}\times 1\right)$ and $y_{mi}\left(n_{2i}\times 1\right)$ are vectors of the observed and missing components of responses in $y_{i}$ satisfying $n_{1i}+n_{2i}=n_{i}$, respectively; $x_{oij}\left(q_{1ij}\times 1\right)$ and $x_{mij}\left(q_{2ij}\times 1\right)$ are vectors of the observed and missing covariate in $x_{ij}$ satisfying $q_{1ij}+q_{2ij}=q$, respectively. Let $r_{yi}={\left(r_{yi1},\ldots ,{r_{y}}_{in_{i}}\right)}^{T}$ be an indicator variable which indicates whether $y_{i}={\left(y_{i1},\ldots ,y_{in_{i}}\right)}^{T}$ is missing; that is,

$$r_{yij}=\left\{\begin{array}{c} 1,\;y_{ij}\;\mathrm{is}\;\mathrm{missing},\\ 0,\;y_{ij}\;\mathrm{is}\;\mathrm{observed}. \end{array}\right.$$

Inspired by Ibrahim et al. [14], it is common to specify a Bernoulli distribution for the following nonignorable missing mechanism. Thus, given $y_{i}$ and unknown parameter $\varphi_{y}$, the conditional probability function of $r_{yij}$ is distributed as

$$p\left(r_{yij}|y_{i},\varphi_{y}\right)={\left\{\mathrm{Pr}(r_{yij}=1|y_{i},\varphi_{y})\right\}}^{r_{y_{ij}}}{\left\{1-\mathrm{Pr}(r_{yij}=1|y_{i},\varphi_{y})\right\}}^{1-r_{y_{ij}}},$$

where $\mathrm{Pr}(r_{yij}=1|y_{i},\varphi_{y})$ is specified by a logistic regression model,

(8) $$\mathrm{logit}\left\{\mathrm{Pr}(r_{yij}=1|y_{i},\varphi_{y})\right\}=\varphi_{y0}+\varphi_{y1}y_{ij}+\varphi_{y2}y_{i,j-1}=u_{yij},$$

in which $\mathrm{logit}\left\{\mathrm{Pr}(r_{yij}=1|y_{i},\varphi_{y})\right\}=\log\left\{\frac{\mathrm{Pr}(r_{yij}=1|y_{i},\varphi_{y})}{1-\mathrm{Pr}(r_{yij}=1|y_{i},\varphi_{y})}\right\}$.

Similarly, let $r_{xij}={\left(r_{xij1},\ldots ,r_{xijq}\right)}^{T}$ be an indicator variable, which indicates whether $x_{ij}$ is missing, and each $r_{xijk}$ is defined as follows:

$$r_{xijk}=\left\{\begin{array}{c} 1,\;x_{ijk}\;\mathrm{is}\;\mathrm{missing},\\ 0,\;x_{ijk}\;\mathrm{is}\;\mathrm{observed}. \end{array}\right.$$

For conditional probability density $\mathrm{Pr}(r_{xij}|x_{ij},\varphi_{x})$, we consider the following nonignorable data mechanisms,

$$\begin{array}{c} \mathrm{Pr}\left(r_{xij}|x_{ij},\varphi_{x}\right)=\mathrm{Pr}\left(r_{xijq}|x_{ij1},\ldots x_{ijq},r_{xij1},\ldots ,r_{xij,q-1},\varphi_{xq}\right)\\ \;\;\;\;\times \mathrm{Pr}(r_{xij,q-1}|x_{ij1},\ldots x_{ij,q-1},r_{xij1},\ldots ,r_{xij,q-2},\varphi_{x,q-1})\\ \;\;\;\;\times \cdots \times \mathrm{Pr}\left(r_{xij2}|x_{ij1},x_{ij2},r_{xij1},\varphi_{x2}\right)\mathrm{Pr}\left(r_{xij1}|x_{ij1},\varphi_{x1}\right), \end{array}$$

in which $\mathrm{Pr}(r_{xijk}|x_{ij1},\ldots ,x_{ijk},r_{xij1},\ldots ,r_{xij,k-1},\varphi_{xk})$ is defined by a logistic regression model

(9) $$\begin{array}{c} \mathrm{logit}\left\{Pr(r_{xijk}=1|x_{ij1},\ldots ,x_{ijk},r_{xij1},\ldots ,r_{xij,k-1},\varphi_{xk})\right\}\\ \;=\varphi_{xk0}+\varphi_{xk1}x_{ij1}+\cdots +\varphi_{xkk}x_{ijk}+\varphi_{xk,k+1}r_{xij1}+\cdots +\varphi_{xk,2k-1}r_{xij,k-1}=v_{xijk}, \end{array}$$

where $\varphi_{xk}={\left(\varphi_{xk0},\varphi_{xk1},\ldots ,\varphi_{xk,2k-1}\right)}^{T}$.

In this article, we consider the following other type of the nonignorable missing mechanism for response and covariates. Specifically, in the first type, the nonignorable missing mechanism for response is specified by a logistic regression model,

(10) $$\mathrm{logit}\left\{\mathrm{Pr}(r_{yij}=1|y_{i},\varphi_{y},x_{ij})\right\}=\varphi_{y0}+\varphi_{y1}y_{ij}+\varphi_{y2}x_{ij1}+\varphi_{y3}x_{ij2}+\cdots +\varphi_{y,k+1}x_{ijk},$$

where $x_{ij1},\ldots x_{ijk}$ are all missing covariables. For missing covariate, $\mathrm{Pr}(r_{xijk}|x_{ij1},\ldots ,x_{ijk},y_{ij},\varphi_{xk})$ is given by a logistic regression model,

(11) $$\mathrm{logit}\left\{\mathrm{Pr}(r_{xijk}=1|x_{ij1},\ldots ,x_{ijk},y_{ij},\varphi_{xk})\right\}=\varphi_{xk0}+\varphi_{xk1}x_{ij1}+\cdots +\varphi_{xkk}x_{ijk}+\varphi_{xk,k+1}y_{ij}=v_{xijk},$$

where $\varphi_{xk}={\left(\varphi_{xk0},\varphi_{xk1},\ldots ,\varphi_{xk,k+1}\right)}^{T}$.

In what follows, we assume that the covariate $x_{ij}={\left(x_{ij1},x_{ij2},\ldots ,x_{ijq}\right)}^{T}$ is continuous, and there is missingness in the first *m* dimension and complete observation in the rest q−m dimension. According to Ibrahim et al. [13], the joint probability function of the missing covariates is simplied by a sequence of one-dimensional conditional distributions as follows,

(12) $$\begin{array}{c} p\left(x_{ij1},x_{ij2},\ldots ,x_{ij,m-1},x_{ijm}|x_{0},\alpha \right)=p\left(x_{ijm}|x_{0},x_{ij1},x_{ij2},\ldots ,x_{ij,m-1},\alpha_{m}\right)\\ \;\;\;\;\times \ldots \times p\left(x_{ij2}|x_{0},x_{ij1},\alpha_{2}\right),p\left(x_{ij1}|x_{0},\alpha_{1}\right) \end{array},$$

where i=1,…,n and $j=1,\ldots ,n_{i}$, $x_{0}=\left(x_{ij,m+1},x_{ij,m+2},\ldots ,x_{ijq}\right)$, $\alpha =(\alpha_{1},\alpha_{2},\ldots ,\alpha_{m})$. Here, covariates $x_{0}$ do not need to be modelled because they are always observed. In addition, continuous missing covariates are generally assumed to follow the normal distribution. For example,

(13) $$p\left(x_{ijk}|x_{0},x_{ij1},x_{ij2},\ldots ,x_{ij,k-1},\alpha_{k}\right)∼N\left(\mu_{ijk},\sigma_{k}^{2}\right),k=1,2,\ldots ,m,$$

where mean parameter $\mu_{ijk}$ is given by

$$\begin{array}{c} \mu_{ijk}=\alpha_{k,0}+\alpha_{k1}x_{ij1}+\alpha_{k2}x_{ij2}+\cdots +\alpha_{k,k-1}x_{ij,k-1}+\alpha_{kk}x_{ij,k+1}\\ \;\;+\alpha_{k,k+1}x_{ij,k+2}+\cdots +\alpha_{k,q-1}x_{ijq}. \end{array}$$

Here, $\alpha_{k}=\left(\alpha_{k,0},\alpha_{k1},\ldots ,\alpha_{k,q-1}\right).$

## 4. Bayesian Inference

To investigate the Bayesian inference on parameters of interest, we first introduce the following notations. Let $Y_{o}=\left\{y_{o1},\ldots ,y_{on}\right\}$ and $Y_{m}=\left\{y_{m1},\ldots ,y_{mn}\right\}$ be the sets of observed and missing values of response variables, respectively. Similarly, $X_{o}=\left\{x_{o11},\ldots ,x_{o1n_{i}},\ldots ,x_{on1},\ldots ,x_{onn_{n}}\right\}$ and $X_{m}=\left\{x_{m11},\ldots ,x_{m1n_{i}},\ldots ,x_{mn1},\ldots ,x_{mnn_{n}}\right\}$ are the sets of observed and missing values corresponding to covariates, respectively. Let $U=\{u_{ij}:i=1,\ldots ,n,j=1,\ldots ,n_{i}\}$ denote the latent variable. Let $b=\{b_{1},\ldots ,b_{n}\}$ and $Z=\{z_{ij}:i=1,\ldots ,n,j=1,\ldots ,n_{i}\}$ denote the vector of random effects and the vector of covariates relating to random effects. Let $T=\{t_{ij}:i=1,\ldots ,n,j=1,\ldots ,n_{i}\}$ be the vector of time effects relating to the nonparametric part. Denote the vector of indicator variables and parameters relating to missing data mechanism by $r=\{r_{y},r_{x}\}$ and $\varphi =\{\varphi_{x},\varphi_{y}\}$, where $r_{y}=\left\{r_{y1},\ldots ,r_{yn}\right\}$ and $r_{x}=\left\{r_{x11},\ldots ,r_{x1n_{1}},\ldots ,r_{xn1},\ldots ,r_{xnn_{n}}\right\}$. On the whole, let $\theta =\{\beta ,p,\phi ,\Sigma ,\xi ,\tau_{\xi }^{2},\delta ,\varphi \}$, α, and $\sigma_{m}^{2}=\left\{\sigma_{xk}^{2}:k=1,\ldots ,m\right\}$ be all the parameters to be estimated in our considered model. Given the observed data $\left\{Y_{o},X_{o},Z,T,r\right\}$, the joint posterior distribution of $\theta ,\alpha ,\sigma_{m}^{2}$ is given by

(14) $$\begin{array}{c} p(\theta ,\alpha ,\sigma_{m}^{2}|Y_{o},X_{o},Z,,T,r)\\ \;\propto p\left(Y_{o}|X_{o},Z,T,\theta \right)p\left(r|Y_{o},X_{o},\varphi \right)p\left(X_{o}|\alpha ,\sigma_{m}^{2}\right)p\left(\theta \right)p\left(\alpha \right)p\left(\sigma_{m}^{2}\right), \end{array}$$

where $p\left(Y_{o}|X_{o},Z,T,\beta ,p,\phi ,\xi \right)=\int p(Y,U,b|X,Z,T,\beta ,p,\phi ,\xi )dY_{m}dX_{m}dUdb$ and $p\left(r|Y_{o},X_{o},\varphi \right)=\int p(r|Y,X,\varphi )dY_{m}dX_{m}$.

Clearly, it is difficult to generate the random sample from the posterior distribution $p(\theta ,\alpha ,\sigma_{m}^{2}|Y_{o},X_{o},Z,T,r)$ because Equation (14) has high-dimensional integration. Thus, inspired by the data augmentation method (Tanner and Wong [23]), we adopt the following posterior distribution, $p(\theta ,\alpha ,\sigma_{m}^{2}|Y,X,T,Z,r,b,U)$, to solve the high-dimensional integration issue. Meanwhile, it is easy to generate the random sample from $p(Y_{m},X_{m},b,U,\theta ,\alpha ,\sigma_{m}^{2}|Y_{o},X_{o},Z,T,r)$ via the Gibbs sampler (Geman and Geman [24]). That is, random samples $\left\{Y_{m},X_{m},U,b,\theta ,\alpha ,\sigma_{m}^{2}\right\}$ are iteratively generated by means of the following conditional distributions $p(Y_{m}|Y_{o},X,U,Z,T,b,r,\theta )$, $p(X_{m}|Y,X_{o},U,Z,T,b,r,\varphi_{x},\alpha ,\sigma_{m}^{2})$,p(U|Y,X,Z,T,p,ϕ), p(b|Y,X,U,Z,T,θ), p(β,p,ϕ,∑,ξ|Y,X,U,Z,T),$p(\tau_{\xi }^{2}|\xi ,\delta )$,$p(\delta_{l}|\xi ,\tau_{\xi }^{2})$, p(φ|Y,X,r), $p(\alpha |X,\sigma_{m}^{2})$, and $p(\sigma_{m}^{2}|X,\alpha )$. To derive the abovementioned conditional distributions, we adopt the following joint logarithmic likelihood function of (Y,U,b)

(15) $$\begin{array}{cc} l(\theta ;Y,U,b) & =\log\left\{\prod_{i=1}^{n}\prod_{j=1}^{n_{i}}p(y_{ij},u_{ij}|b_{i},\theta )p\left(b_{i}\right)\right\}\\ \; & =\sum_{y_{ij}=0}\log p(y_{ij},u_{ij}|,b_{i},\theta )+\sum_{y_{ij}>0}\log p(y_{ij},u_{ij}|b_{i},\theta )+\sum_{i=1}^{n}\log\left(p\left(b_{i}\right)\right)\\ \; & =-\frac{1}{\phi }\sum_{i=1}^{n}\sum_{j=1}^{n_{i}}\frac{\mu_{ij}^{2-p}}{2-p}-\sum_{y_{ij}>0}\left(\frac{y_{ij}}{\phi \left(p-1\right)\mu_{ij}^{p-1}}+\log\Gamma \left(u_{ij}\frac{2-p}{p-1}\right)+\log u_{ij}!+\log y_{ij}\right)\\ \; & +\sum_{y_{ij}>0}u_{ij}\left(\frac{2-p}{p-1}\log\frac{y_{ij}}{p-1}-\frac{\log\phi }{p-1}-\log\left(2-p\right)\right)+\sum_{i=1}^{n}\log\left(p\left(b_{i}\right)\right) \end{array}.$$

Moreover, the prior distributions of β, *p*, ϕ, Σ, ξ, $\tau_{\xi }^{2}$, $\delta_{\rho }$, φ, α and $\sigma_{xk}^{2}$ are given by

(16) $$\begin{array}{c} p\left(\beta \right)∼N\left(\beta^{0},A\right),\mathrm{logit}\left(p-1\right)∼N\left(0,10000\right),\log\left(\phi \right)∼N\left(0,10000\right),p\left(\Sigma \right)∼IW_{k}\left(\rho_{0},R_{0}\right),\\ \xi |\tau_{\xi }^{2}\propto \exp\left(-\frac{1}{2\tau_{\xi }^{2}}\xi^{T}Q\xi \right),p\left(\tau_{\xi }^{2}\right)∼IG\left(a_{\tau },b_{\tau }\right),p\left(\delta_{\rho }\right)∼\Gamma \left(a_{\delta },b_{\delta }\right),p\left(\varphi_{y}\right)∼N\left(\varphi_{y}^{0},B\right),\\ p\left(\varphi_{x}\right)∼N\left(\varphi_{x}^{0},C\right),p\left(\alpha \right)∼N\left(\alpha_{x}^{0},D\right),p\left(\sigma_{xk}^{2}\right)∼IG\left(a_{xk},b_{xk}\right), \end{array}$$

where $\rho_{0},a_{\tau },b_{\tau },a_{\delta },b_{\delta },a_{xk},b_{xk},\varphi_{x}^{0},\varphi_{y}^{0},\alpha^{0},A,B,C,D,\Sigma ,R_{0}$ is the pregiven hyperparameter, *N* is the normal distribution, $IW_{k}\left(\cdot ,\cdot \right)$ is the k-dimensional inverse Wishart distribution, Γ is the gamma distribution, *IG* is the inverse gamma distribution, and $\mathrm{logit}\left(a\right)=\log\left(\frac{a}{1-a}\right)$. As for the choices of hyperparameters with regard to the Bayesian P-spline method, Lang and Brezger [22] pointed out that $a_{\tau }=1$ and a small value for $b_{\tau }$ for example, $b_{\tau }=0.005$ or $b_{\tau }=0.0005$, leading to an almost diffuse prior for $\tau_{\xi }^{2}$. Moreover, the hyperparameters $a_{\delta }$ and $b_{\delta }$ are simultaneously taken to be 0.5, which can characterize the highly oscillating features for some nonparametric functions. As for the power parameter *p*, Ye et al. [12] adopted the following priors to conduct the sensitivity analysis: p∼Uniform(1,2), logit(p−1)∼N(0,100), p−1∼Beta(0.1,0.1) and p−1∼Beta(0.01,0.01). As a result, Ye et al. [12] chose the logit(p−1)∼N(0,100) prior as the optimal for *p* in the Tweedie compound Poisson distribution based on the sensitivity analysis. The choices of hyperparameters for other prior distributions are discussed in Section 5. The conditional distributions, Gibbs sampling and Metropolis–Hastings algorithm are shown in the Appendix A and Appendix B.

### Bayesian Estimates

Let $\left\{\beta^{\left(l\right)},\phi^{\left(l\right)},p^{\left(l\right)},\Sigma^{\left(l\right)},\alpha^{\left(l\right)},\varphi_{y}^{\left(l\right)},\varphi_{x}^{\left(l\right)},\sigma {{}_{xk}^{2}}^{\left(l\right)}:l=1,\ldots ,L\right\}$ be random samples from the joint posterior distribution $p(\beta ,\phi ,p,\Sigma ,\alpha ,\varphi_{y},\varphi_{x},\sigma_{xk}^{2}|Y,X,Z,r,b)$. The Bayesian estimates of parameters β, ϕ, *p*, Σ, α,$\varphi_{y}$,$\varphi_{x}$ and $\sigma_{xk}^{2}$ can be obtained by

$$\begin{array}{c} \hat{\beta }=\frac{1}{L}\sum_{l=1}^{L}\beta^{\left(l\right)},\hat{\phi }=\frac{1}{L}\sum_{l=1}^{L}\phi^{\left(l\right)},\hat{p}=\frac{1}{L}\sum_{l=1}^{L}p^{\left(l\right)},\hat{\Sigma }=\frac{1}{L}\sum_{l=1}^{L}\Sigma^{\left(l\right)},\\ \hat{\alpha }=\frac{1}{L}\sum_{l=1}^{L}\alpha^{\left(l\right)},{\hat{\varphi }}_{y}=\frac{1}{L}\sum_{l=1}^{L}\varphi_{y}^{\left(l\right)},{\hat{\varphi }}_{x}=\frac{1}{L}\sum_{l=1}^{L}\varphi_{x}^{\left(l\right)},{\hat{\sigma }}_{xk}^{2}=\frac{1}{L}\sum_{l=1}^{L}\sigma {{}_{xk}^{2}}^{\left(l\right)} \end{array}.$$

Similarly, the consistency estimates of the posterior covariance matrix $\mathrm{var}(\beta ,\phi ,p,\Sigma ,\alpha ,\varphi_{y},\varphi_{x},\sigma_{xk}^{2}|Y,X,Z,r,b)$ for parameters β, ϕ, *p*, Σ, α,$\varphi_{y}$,$\varphi_{x}$ and $\sigma_{xk}^{2}$ can be obtained from the sample covariance matrix of their random samples. For example, the posterior covariance matrix var(β|Y,X,Z,r,b) can be consistently estimated by

$$\hat{\mathrm{var}}\left(\beta |Y,X,Z,r,b\right)=\frac{1}{L-1}\sum_{l=1}^{L}\left(\beta^{\left(l\right)}-\hat{\beta }\right){\left(\beta^{\left(l\right)}-\hat{\beta }\right)}^{T}.$$

In addition, the corresponding standard deviation can be estimated by the diagonal elements of the sample covariance matrix of the random sample sequence.

## 5. Numerical Examples

In this section, two simulation studies and a real example relating to the OAI data are conducted to investigate the performance of our proposed Bayesian methodologies.

### 5.1. Simulation Studies

In the first simulation study, we assume that the longitudinal semicontinuous datasets $\left\{y_{ij}:i=1,\ldots ,n,j=1,\ldots n_{i}\right\}$ with n=150 and $n_{i}=4$ are simulated from the Tweedie compound Poisson distribution ${\mathrm{Tw}}_{p}\left(\mu_{ij},\phi \right)$ and the conditional mean $\mu_{ij}$ is given by

(17) $$\log\left(\mu_{ij}\right)=x_{ij1}\beta_{1}+x_{ij2}\beta_{2}+x_{ij3}\beta_{3}+b_{i}+g\left(t_{ij}\right),$$

where covariate $x_{ij3}$ is generated from the standard normal distribution, and $x_{ij1}$ and $x_{ij2}$ are independently simulated from the normal distribution $N(\alpha_{10}+\alpha_{11}x{}_{ij3},\sigma_{x1}^{2})$ and $N(\alpha_{20}+\alpha_{21}x_{ij1}+\alpha_{22}x_{ij3},\sigma_{x2}^{2})$, respectively. In addition, the random effects $b_{i}$ are independent and identically distributed as N(0,Σ), and the true curve of nonparametric function is given by g(t)=sin(2πt) with $t_{ij}∼U\left(0,1\right)$. The true values of the abovementioned parameters are taken to be $\sigma_{x1}^{2}=0.25$, $\sigma_{x2}^{2}=0.36$, p=1.5, ϕ=0.5, Σ=0.64, $\beta ={\left(\beta_{1},\beta_{2},\beta_{3}\right)}^{T}={\left(1,1,-1\right)}^{T}$, $\alpha_{1}={\left(\alpha_{10},\alpha_{11}\right)}^{T}={\left(0.05,0.5\right)}^{T}$, $\alpha_{2}={\left(\alpha_{20},\alpha_{21},\alpha_{22}\right)}^{T}={\left(-0.9,0.05,0.9\right)}^{T}$. In what follows, it is assumed that covariate $x_{ij3}$ is completely observed, while response $y_{ij}$ and covariates $x_{ij1}$, $x_{ij2}$ are subject to missingness. Thus, the nonignorable missing mechanism for these three variables are modelled by the following logistic regression model,

(18) $$\begin{array}{c} \mathrm{logit}\left\{\mathrm{Pr}(r_{yij}=1|y_{ij},y_{i,j-1},\varphi_{y})\right\}=\varphi_{y0}+\varphi_{y1}y_{ij}+\varphi_{y2}y_{i,j-1},\\ \mathrm{logit}\left\{\mathrm{Pr}(r_{xij1}=1|x_{ij1},\varphi_{x1})\right\}=\varphi_{x10}+\varphi_{x11}x_{ij1},\\ \mathrm{logit}\left\{\mathrm{Pr}(r_{xij2}=1|x_{ij1},x_{ij2},r_{xij1},\varphi_{x2})\right\}=\varphi_{x20}+\varphi_{x21}x_{ij1}+\varphi_{x22}x_{ij2}+\varphi_{x23}r_{xij1}, \end{array}$$

where the truth values of $\varphi_{y}$, $\varphi_{x1}$, and $\varphi_{x2}$ are given by $\varphi_{y}={\left(\varphi_{y0},\varphi_{y1},\varphi_{y2}\right)}^{T}={\left(-2.4,0.1,0.1\right)}^{T}$, $\varphi_{x1}={\left(\varphi_{x10},\varphi_{x11}\right)}^{T}={\left(-2.5,0.1\right)}^{T}$, $\varphi_{x2}={\left(\varphi_{x20},\varphi_{x21},\varphi_{x22},\varphi_{x23}\right)}^{T}={\left(-1.9,0.05,0.05,0.3\right)}^{T}$. The missing data for $x_{ij1}$, $x_{ij2}$ and $y_{ij}$ were generated by (18), and the average proportion of missing data for $x_{ij1}$, $x_{ij2}$ and $y_{ij}$ on the basis of 50 replications are 8.7%, 13.3%, and 7.5%, respectively.

To investigate the effect of different prior information on the Bayesian estimate for unknown parameters, three types of prior information are considered as follows.

Type I: The hyperparameters $\beta^{0}$, $\varphi_{y}^{0}$, $\varphi_{x1}^{0}$, $\varphi_{x2}^{0}$, $\alpha_{x1}^{0}$ and $\alpha_{x2}^{0}$ are taken to be the truth values corresponding to their parameters; $\rho_{0}=8$, $R_{0}=2$, $a_{\tau }=1$, $b_{\tau }=0.005$, $a_{\delta }=0.5$ and $b_{\delta }=0.5$; ***A***, ***B***, $C_{x1}$, $C_{x2}$, $D_{x1}$, and $D_{x2}$ are taken to be $0.25I_{3}$, $0.25I_{3}$, $0.25I_{2}$, $0.25I_{4}$, $0.25I_{2}$, $0.25I_{3}$, where $I_{d}$ denotes the d×d identity matrix. This scenario is viewed as a good piece of prior information.

Type II: The hyperparameters $\beta^{0}$, $\varphi_{y}^{0}$, $\varphi_{x1}^{0}$, $\varphi_{x2}^{0}$, $\alpha_{x1}^{0}$ and $\alpha_{x2}^{0}$ are taken to be 2 times truth values corresponding to their parameters; ***A***, ***B***, $C_{x1}$, $C_{x2}$, $D_{x1}$, and $D_{x2}$ are taken to be $0.75I_{3}$, $0.75I_{3}$, $0.75I_{2}$, $0.75I_{4}$, while other hyperparameters are taken to be the same as those given in Type I. This scenario is viewed as an inaccurate prior information.

Type III: The hyperparameters $\beta^{0}$, $\varphi_{y}^{0}$, $\varphi_{x1}^{0}$, $\varphi_{x2}^{0}$, $\alpha_{x1}^{0}$, and $\alpha_{x2}^{0}$ are taken to be zero vector, respectively; ***A***, ***B***, $C_{x1}$, $C_{x2}$, $D_{x1}$, and $D_{x2}$ are taken to be $100I_{3}$, $100I_{3}$, $100I_{2}$, $100I_{4}$, while other hyperparameters are taken to be the same as those given in Type I. This scenario is viewed as a noninformative prior information.

**Figure 2:**
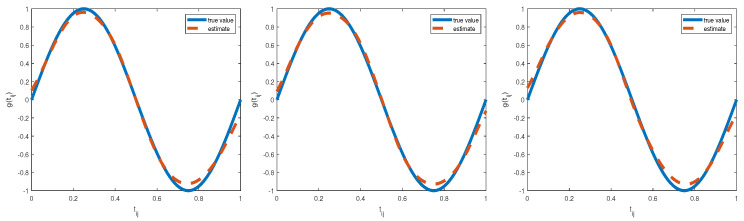
The estimated function and true function of g(t) for three priors: type I (**left panel**), type II (**middle panel**), and type III (**right panel**) in the first simulation.

For each of the above-generated 50 datasets, the hybrid algorithm combining the block Gibbs sampler and Metropolis–Hastings algorithm is used to produce the joint Bayesian estimates of unknown parameters, random effects, and nonparametric function. To ensure the convergence of the hybrid algorithm for each replication, we collected 5000 observations after 5000 iterations to calculate Bayesian estimates, which are reported in Table 3, where “Bias” is the difference between the mean value of parameters obtained from 50 replication and the truth value, “SD” is the standard deviation of the estimates on the basis of 50 replications, and “RMS” is the root mean square between the estimates on the basis of 50 replications and its true value. It can be seen from Table 3 that (i) Bayesian estimates for unknown parameters were reasonably accurate in our considered three different prior information because all Bias values are less than 0.1, and (ii) the estimated values of SD and RMS are less than 0.5 and there is little difference between these two estimated values regardless of any priors. Thus, Bayesian estimates are not sensitive to our considered three prior pieces of information. In addition, examination of Figure 2 indicated that our proposed Bayesian P-spline method to approximate nonparametric function is validated to be feasible because the estimated curves of nonparametric function g(t) matched well with the true curve in our considered simulation studies.

**Figure 3:**
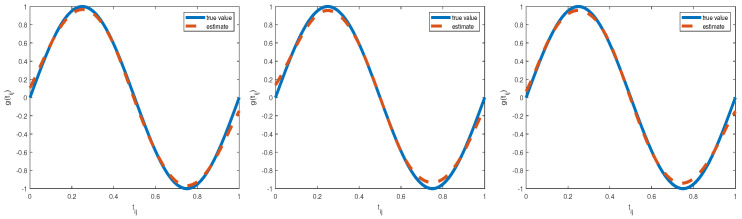
The estimated function and true function of g(t) for three priors: type I (**left panel**), type II (**middle panel**), type III (**right panel**) in the second simulation.

In the second simulation study, the simulated setup is the same as the first simulation study except for the missing mechanism. Here, the other nonignorable missing mechanism model for $y_{ij}$, $x_{ij1}$ and $x_{ij2}$ is given by

(19) $$\begin{array}{c} \mathrm{logit}\left\{\mathrm{Pr}(r_{yij}=1|y_{ij},x_{ij1},x_{ij2},\varphi_{y})\right\}=\varphi_{y0}+\varphi_{y1}y_{ij}+\varphi_{y2}x_{ij1}+\varphi_{y3}x_{ij2},\\ \mathrm{logit}\left\{\mathrm{Pr}(r_{xij1}=1|x_{ij1},y_{ij},\varphi_{x1})\right\}=\varphi_{x10}+\varphi_{x11}x_{ij1}+\varphi_{x12}y_{ij},\\ \mathrm{logit}\left\{\mathrm{Pr}(r_{xij2}=1|x_{ij1},x_{ij2},y_{ij},\varphi_{x2})\right\}=\varphi_{x20}+\varphi_{x21}x_{ij1}+\varphi_{x22}x_{ij2}+\varphi_{x23}y_{ij} \end{array},$$

where the true values of unknown parameters are taken to be $\varphi_{y}={\left(\varphi_{y0},\varphi_{y1},\varphi_{y2},\varphi_{y3}\right)}^{T}={\left(-2.2,0.1,0.1,0.1\right)}^{T}$, $\varphi_{x1}={\left(\varphi_{x10},\varphi_{x11},\varphi_{x12}\right)}^{T}={\left(-2.5,0.2,0.1\right)}^{T}$, $\varphi_{x2}={\left(\varphi_{x20},\varphi_{x21},\varphi_{x22},and\varphi_{x23}\right)}^{T}={\left(-1.9,0.05,0.05,-0.3\right)}^{T}$. The average proportion of missing data for $x_{ij1}$, $x_{ij2}$ and $y_{ij}$ is 10%, 11%, and 8.5%, respectively. Similar to the first simulation study, we also considered three different prior pieces of information for their corresponding hyperparameters. These findings in Table 4 and Figure 3 show that (i) all Bias values corresponding to unknown parameters are less than 0.1 except that the Bias value for $\varphi_{x10}$ is −0.1013 under the Type II prior and the Bias values for $\varphi_{y0}$ and $\varphi_{x23}$ are −0.1102 and −0.1029 under the Type III prior, respectively, and (ii) the estimated curves of nonparametric function g(t) also matched well with the true curve regardless of three different priors. Clearly, Bayesian estimates under the first two priors are better than those obtained from the Type III prior. All in all, our proposed Bayesian approach is feasible in our considered missing mechanism.

### 5.2. Real Example

In this section, the application of our proposed semiparametric Bayesian approach is illustrated by the analysis of longitudinal semicontinuous data from the OAI, which was discussed in Section 2. The OAI longitudinal data were analyzed by various approaches, such as Chen and Wehrly [25,26]. However, these authors only considered the observed data by reducing 4796 patients to 1499 patients and assumed the log transformation of the WOMAC score plus 1 to approximate the normal distribution. In this study, our scientific interest is to link the covariates, such as age, sex, and BMI with the outcome WOMAC score while accounting for nonignorable missing response with a point mass at zero and covariates data. In addition, we viewed age as the individual-level covariate modeled nonparametrically with the other covariate variables modeled parametrically. Let the outcome $Y_{ij}$ represent the WOMAC numeric score for the right knees of the *i*th (i=1,2,…,4796) patient recorded at the *j*th time point (j=1,2,…,5 corresponding to 0, 12, 24, 36, and 48 months). As discussed in Section 2, we regarded the WOMAC numerical score as a longitudinal semicontinuous outcome in this real example.

**Figure 4:**
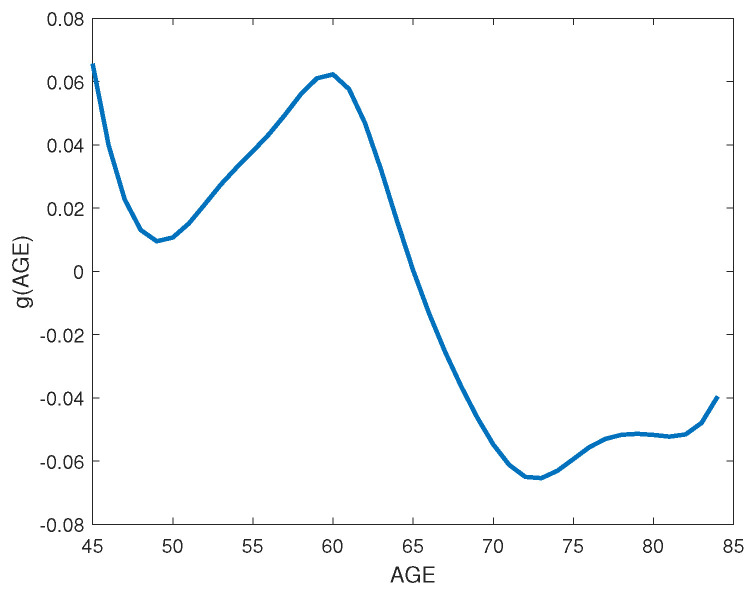
Nonparametric estimate of effects of age on the WOMAC numeric score in the OAI dataset.

Here, given random effects $b_{i}$, $Y_{ij}|b_{i}$ follows the Tweedie compound Poisson distribution; that is $Y_{ij}|b_{i}∼{\mathrm{Tw}}_{p}\left(\mu_{ij},\phi \right)$. The conditional mean $\mu_{ij}$ is simultaneously linked to covariates, random effects, and nonparametric function as follows,

$$\log\left(\mu_{ij}\right)=\beta_{0}+\beta_{1}{\mathrm{BMI}}_{ij}+\beta_{2}{\mathrm{SEX}}_{ij}+b_{i}+g\left({\mathrm{AGE}}_{ij}\right),$$

where the covariates ${\mathrm{SEX}}_{ij}$ (1 for male or 2 for female) and ${\mathrm{AGE}}_{ij}$ are completely observed, while the outcome $Y_{ij}$ and covariate ${\mathrm{BMI}}_{ij}$ are missing and their corresponding missing rates are 8.5% and 13.5%, respectively. Furthermore, we consider the following missing data mechanisms for covariate ${\mathrm{BMI}}_{ij}$ and outcome $Y_{ij}$,

$$\begin{array}{c} \mathrm{logit}\left\{\mathrm{Pr}(r_{{\mathrm{BMI}}_{ij}}=1|{\mathrm{BMI}}_{ij},\varphi_{BMI})\right\}=\varphi_{BMI0}+\varphi_{BMI1}{\mathrm{BMI}}_{ij},\\ \mathrm{logit}\left\{\mathrm{Pr}(r_{Y_{ij}}=1|Y_{ij},Y_{i,j-1},\varphi_{Y})\right\}=\varphi_{Y0}+\varphi_{Y1}Y_{ij}+\varphi_{Y2}Y_{i,j-1}, \end{array}$$

where $\varphi_{BMI}={\left(\varphi_{BMI0},\varphi_{BMI1}\right)}^{T}$, and $\varphi_{Y}={\left(\varphi_{Y0},\varphi_{Y1},\varphi_{Y2}\right)}^{T}$. In addition, we assume that the missing distribution for covariate ${\mathrm{BMI}}_{ij}$ follows the normal distribution $N(\alpha_{10}+\alpha_{11}{\mathrm{SEX}}_{ij},\sigma_{BMI}^{2})$ and random effect $b_{i}$ is distributed as the normal distribution N(0,Σ). Bayesian estimates of unknown parameters and their corresponding standard error as well as the nonparametric function are displayed in Table 5 and Figure 4. Table 5 indicates that the covariates BMI and Sex have the positive significant effect on the WOMAC score at the significance level of 0.05. The result shows that the WOMAC score increases as BMI increases. The higher the BMI score a patient has, the greater intensity of knee osteoarthritis the patient will suffer. The positive significant effect of the covariate Sex on the WOMAC score indicates that the average WOMAC score for females are higher compared with males. Women are more vulnerable to greater intensity than men. Chen and Wehrly [25,26] assumed a linear age effect on the WOMAC score parametrically, but an insignificant effect on the WOMAC score are presented in their studies. It appears from Figure 4 that the Bayesian estimates of nonparametric function $g({\mathrm{AGE}}_{ij})$ based on the P-spline method has a significant nonlinear trend. Specifically, there was a sharp decrease from age 45 to approximately age 49 and from age 60 to approximately age 73, respectively. Moreover, stabilization seems to have started at age 73. In the missing mechanism model, we found that the Bayesian estimates of unknown parameters $\varphi_{BMI1}$ and $\varphi_{Y2}$ significantly deviated from zero. Thus, it is reasonable to incorporate the missing data into our proposed semiparametric Bayesian model in the analysis of OAI dataset because missing data mechanisms for $Y_{ij}$ and ${\mathrm{BMI}}_{ij}$ are nonignorable.

## 6. Conclusions

In this paper, we have introduced a new Tweedie compound Poisson partial linear mixed model with nonignorable missing covariates and responses by assuming that the random effect is distributed as a multivariate normal distribution and the nonparametric function is modelled by the Bayesian P-splines simultaneously. The logistic regression model is simultaneously used to model the missing response and covariate mechanisms. This article has the following contributions: (i) our proposed Bayesian semiparametric mixed effects model can model both zero and positive components of the longitudinal semicontinuous data in an integral way while accounting for the nonignorable missing responses and covariates simultaneously; (ii) our proposed partial linear mixed models based on Bayesian P-spline can characterize the nonlinear smooth effects of covariate in the analysis of longitudinal semicontinuous data; (iii) the conditional distributions for the Gibbs sampling algorithm and Metropolis–Hastings algorithm of our proposed model are derived; and (iv) two simulation studies and a real example are used to illustrate the effectiveness and feasibility of our several considered missing mechanisms.

## Figures and Tables

**Table 1:** Symbols and description.

Symbols	Description
*U*	A Poisson distribution random variable
μ	The mean parameter of Tweedie compound Poisson distribution
ϕ	The dispersion parameter of Tweedie compound Poisson distribution
*p*	The power parameter of Tweedie compound Poisson distribution
β	A q×1 vector of unknown regression parameter
$b_{i}$	A d×1 vector of random effect $\left(b_{i}∼N_{d}\left(0,\Sigma \right)\right)$
Σ	A d×d covariance matrix
g(·)	An unknown nonparametric function
ξ	An H×1 vector of B-spline coefficient $(\xi ={\left(\xi_{1},\ldots ,\xi_{H}\right)}^{T}$)
B(·)	The B-spline basis function
$\tau_{\xi }^{2}$	A global smoothing parameter
***Q***	The H×H penalty matrix with elements $\delta ={\left(\delta_{2},\ldots ,\delta_{H}\right)}^{T}$
$Y_{o}$	Set of observed values of response variable *Y* $\left(Y_{o}=\left\{y_{o1},\ldots ,y_{on}\right\}\right)$
$Y_{m}$	Set of missing values of response variable *Y* $\left(Y_{m}=\left\{y_{m1},\ldots ,y_{mn}\right\}\right)$
$X_{o}$	Set of observed values of covariates *X* $\left(X_{o}=\left\{x_{o11},\ldots x_{o1n_{1}},\ldots ,x_{on1},\ldots ,x_{onn_{n}}\right\}\right)$
$X_{m}$	Set of missing values of covariates *X* $\left(X_{m}=\left\{x_{m11},\ldots x_{m1n_{1}},\ldots ,x_{mn1},\ldots ,x_{mnn_{n}}\right\}\right)$
***Z***	Vector of covariates relating to random effects $\left(Z=\left\{z_{ij}:i=1,\ldots ,n,j=1,\ldots n_{i}\right\}\right)$
***T***	Vector of time effects $\left(T=\left\{t_{ij}:i=1,\ldots ,n,j=1,\ldots ,n_{i}\right\}\right)$
***r***	Vector of indicator variables relating to missing data mechanism ($r=\{r_{y},r_{x}\}$)
φ	Vector of parameters relating to missing data mechanism ($\varphi =\{\varphi_{x},\varphi_{y}\}$)
α	The parameter in the covariables’ distribution $\left(\alpha =\left(\alpha_{1},\ldots ,\alpha_{m}\right)\right)$
$\sigma_{m}^{2}$	The parameter in the covariables’ distribution $\left(\sigma_{m}^{2}=\left\{\sigma_{xk}^{2}:k=1,\ldots ,m\right\}\right)$

**Table 2:** Sample data from the OAI study (M denotes the missing data).

ID	Month	Response Variable	Covariates		
		WOMAC Score	BMI	SEX	AGE
9019406	0	0	23.5	Male	71
9019406	12	1	22.6	Male	72
9019406	24	0	22.9	Male	73
9019406	36	M	M	Male	74
9019406	48	M	M	Male	75
⋮	⋮	⋮	⋮	⋮	⋮
9025191	0	1	24.2	Female	55
9025191	12	0	24.7	Female	56
9025191	24	1.06	24.7	Female	57
9025191	36	1	25.4	Female	58
9025191	48	0	25.4	Female	59
⋮	⋮	⋮	⋮	⋮	⋮

**Table 3:** Bayesian estimates of parameters in the first simulation study.

Par.	Type I			Type II			Type III		
	Bias	SD	RMS	Bias	SD	RMS	Bias	SD	RMS
$\beta_{1}$	0.0007	0.0795	0.0787	−0.0068	0.0664	0.0661	0.0042	0.0763	0.0756
$\beta_{2}$	−0.0011	0.0509	0.0504	0.0026	0.0567	0.0562	0.0045	0.0553	0.0549
$\beta_{3}$	0.0004	0.0723	0.0716	−0.0051	0.0678	0.0673	−0.0068	0.0815	0.0810
*p*	−0.0143	0.0265	0.0299	−0.0180	0.0227	0.0288	−0.0205	0.0206	0.0289
ϕ	−0.0247	0.0473	0.0529	−0.0355	0.0408	0.0538	−0.0371	0.0406	0.0547
Σ	−0.0355	0.0974	0.1028	−0.0195	0.0826	0.0841	−0.0354	0.0871	0.0932
$\alpha_{10}$	0.0193	0.0653	0.0675	0.0159	0.0673	0.0685	0.0293	0.0749	0.0797
$\alpha_{11}$	−0.0031	0.0866	0.0858	−0.0148	0.0650	0.0660	0.0023	0.0813	0.0805
$\alpha_{20}$	0.0195	0.0677	0.0698	0.0290	0.0724	0.0773	−0.0021	0.0733	0.0726
$\alpha_{21}$	0.0152	0.1205	0.1203	−0.0075	0.1125	0.1116	0.0041	0.1479	0.1464
$\alpha_{22}$	−0.0171	0.0963	0.0968	0.0144	0.0827	0.0831	−0.0123	0.1036	0.1033
$\varphi_{y0}$	0.0016	0.1453	0.1439	−0.0672	0.1680	0.1794	−0.0565	0.1603	0.1685
$\varphi_{y1}$	−0.0156	0.0592	0.0607	0.0003	0.0468	0.0463	0.0027	0.0539	0.0535
$\varphi_{y2}$	−0.0005	0.0640	0.0633	0.0090	0.0520	0.0522	0.0054	0.0569	0.0566
$\varphi_{x10}$	−0.0119	0.1308	0.1301	−0.0802	0.1660	0.1828	−0.0622	0.1568	0.1673
$\varphi_{x11}$	−0.0275	0.1552	0.1561	0.0095	0.2089	0.2070	0.0126	0.1889	0.1874
$\varphi_{x20}$	−0.0139	0.1512	0.1503	−0.0461	0.2133	0.2162	−0.0886	0.1723	0.1921
$\varphi_{x21}$	−0.0323	0.1915	0.1923	−0.0283	0.1914	0.1915	0.0616	0.2577	0.2624
$\varphi_{x22}$	0.0088	0.1319	0.1309	0.0270	0.1426	0.1437	−0.0375	0.1528	0.1558
$\varphi_{x23}$	−0.0452	0.2707	0.2717	0.0018	0.3127	0.3096	−0.0170	0.4563	0.4521
$\sigma_{x1}^{2}$	0.0200	0.0170	0.0262	0.0245	0.0182	0.0304	0.0215	0.0198	0.0291
$\sigma_{x2}^{2}$	0.0237	0.0233	0.0330	0.0275	0.0214	0.0347	0.0299	0.0220	0.0370

**Table 4:** Bayesian estimation of parameters in the second simulation study.

Par.	Type I			Type II			Type III		
	Bias	SD	RMS	Bias	SD	RMS	Bias	SD	RMS
$\beta_{1}$	−0.0073	0.0794	0.0790	−0.0050	0.0730	0.0724	0.0029	0.0717	0.0711
$\beta_{2}$	−0.0123	0.0551	0.0559	0.0119	0.0560	0.0567	−0.0044	0.0577	0.0573
$\beta_{3}$	0.0121	0.0764	0.0766	−0.0124	0.0786	0.0788	0.0003	0.0711	0.0704
*p*	−0.0182	0.0274	0.0327	−0.0132	0.0255	0.0285	−0.0140	0.0237	0.0273
ϕ	−0.0298	0.0444	0.0531	−0.0262	0.0406	0.0480	−0.0267	0.0416	0.0491
Σ	−0.0343	0.0737	0.0806	−0.0423	0.0904	0.0990	−0.0545	0.0870	0.1019
$\alpha_{10}$	0.0885	0.0785	0.1177	0.0837	0.0595	0.1023	0.0852	0.0748	0.1129
$\alpha_{11}$	0.0057	0.0775	0.0770	0.0180	0.0627	0.0646	0.0106	0.0781	0.0780
$\alpha_{20}$	−0.0466	0.0834	0.0948	−0.0603	0.0661	0.0889	−0.0458	0.0711	0.0840
$\alpha_{21}$	−0.0126	0.1330	0.1322	−0.0493	0.1282	0.1362	−0.0392	0.1376	0.1418
$\alpha_{22}$	0.0065	0.1015	0.1007	0.0071	0.1039	0.1031	0.0001	0.0993	0.0983
$\varphi_{y0}$	0.0094	0.1394	0.1383	−0.0769	0.1987	0.2112	−0.1102	0.1917	0.2194
$\varphi_{y1}$	0.0070	0.0509	0.0508	0.0108	0.0498	0.0505	0.0167	0.0453	0.0479
$\varphi_{y2}$	−0.0251	0.1807	0.1806	−0.0055	0.1977	0.1958	0.0114	0.2291	0.2271
$\varphi_{y3}$	0.0317	0.1131	0.1164	−0.0203	0.1385	0.1386	−0.0355	0.1435	0.1464
$\varphi_{x10}$	−0.0540	0.1440	0.1524	−0.1013	0.1761	0.2016	−0.0449	0.1860	0.1895
$\varphi_{x11}$	−0.0042	0.1922	0.1903	0.0322	0.2089	0.2093	−0.0226	0.2019	0.2011
$\varphi_{x12}$	0.0085	0.0505	0.0507	0.0094	0.0698	0.0697	0.0061	0.0563	0.0561
$\varphi_{x20}$	−0.0337	0.2063	0.2070	−0.0246	0.2234	0.2225	−0.0077	0.2564	0.2540
$\varphi_{x21}$	−0.0478	0.2089	0.2123	0.0187	0.2069	0.2057	0.0169	0.2182	0.2167
$\varphi_{x22}$	0.0007	0.1472	0.1457	−0.0201	0.1322	0.1324	0.0045	0.1744	0.1727
$\varphi_{x23}$	−0.0221	0.1341	0.1346	−0.0780	0.1768	0.1916	−0.1029	0.2064	0.2288
$\sigma_{x1}^{2}$	0.0273	0.0219	0.0349	0.0230	0.0194	0.0300	0.0251	0.0216	0.0330
$\sigma_{x2}^{2}$	0.0353	0.0306	0.0465	0.0336	0.0297	0.0447	0.0364	0.0260	0.0446

**Table 5:** Bayesian estimates and standard errors in the real example.

Parameter	Est	SD
$\beta_{0}$	−0.492835	0.0778
$\beta_{1}$	0.058405	0.0024
$\beta_{2}$	0.141541	0.0370
*p*	1.258745	0.0031
ϕ	3.146945	0.0245
Σ	1.840797	0.0550
$\alpha_{10}$	21.004105	0.3076
$\alpha_{11}$	4.520023	0.1779
$\varphi_{Y0}$	−2.694960	0.0196
$\varphi_{Y1}$	0.000522	0.0024
$\varphi_{Y2}$	0.042958	0.0023
$\varphi_{BMI0}$	−1.338632	0.1937
$\varphi_{BMI1}$	−0.017045	0.0067
$\sigma_{BMI}^{2}$	30.067984	0.5088

## Data Availability

The research data are available on the website: https://www.oai.ucsf.edu, accessed on 4 April 2017.

## Appendix A. Conditional Distributions for the Gibbs Sampling Algorithm

First, the conditional distribution of the missing part used in the Gibbs sampling algorithm is as follows.

(1) The logarithmic joint conditional distribution of ($y_{mi},u_{i})$ is
  $$\begin{array}{cc} \; & \log p(y_{mi},u_{i}|y_{oi},x_{i},z_{i},t_{i},b_{i},\Sigma ,\beta ,p,\phi ,\xi ,r_{yi},\varphi_{y})\\ \; & \propto \log p\left(y_{i},u_{i}|x_{i},z_{i},t_{i},b_{i},\theta \right)+\log p\left(r_{yi}|y_{i},\varphi_{y}\right)\\ \; & =\log\prod_{j=1}^{n_{i}}p\left(y_{ij},u_{ij}|x_{ij},z_{ij},t_{ij},b_{i},\theta \right)+\log\prod_{j=1}^{n_{i}}p(r_{yij}|y_{i},\varphi_{y})\\ \; & =\sum_{j=1}^{n_{i}}\log p\left(y_{ij},u_{ij}|x_{ij},z_{ij},t_{ij},b_{i},\theta \right)+\sum_{j=1}^{n_{i}}\log p\left(r_{yij}|y_{i},\varphi_{y}\right)\\ \; & =-\frac{1}{\phi }\sum_{j=1}^{n_{i}}\frac{\mu_{ij}^{2-p}}{2-p}-\sum_{j=1}^{n_{i}}\left(\frac{y_{ij}}{\phi \left(p-1\right)\mu_{ij}^{p-1}}+\log\Gamma \left(u_{ij}\frac{2-p}{p-1}\right)+\log u_{ij}!+\log y_{ij}\right)I\left\{y_{ij}>0\right\}\\ \; & \;+\sum_{j=1}^{n_{i}}u_{ij}\left(\frac{2-p}{p-1}\log\frac{y_{ij}}{p-1}-\frac{\log\phi }{p-1}-\log\left(2-p\right)\right)I\left\{y_{ij}>0\right\}\\ \; & \;+\sum_{j=1}^{n_{i}}\log\left\{{\left(\frac{\exp(u_{yij})}{1+\exp(u_{yij})}\right)}^{r_{yij}}{\left(1-\frac{\exp(u_{yij})}{1+\exp(u_{yij})}\right)}^{1-r_{yij}}\right\}\\ \; & \propto -\sum_{j=1}^{n_{i}}\left(\frac{y_{ij}}{\phi \left(p-1\right)\mu_{ij}^{p-1}}+\log y_{ij}\right)I\left\{y_{ij}>0\right\}+\sum_{j=1}^{n_{i}}u_{ij}\left(\frac{2-p}{p-1}\log\frac{y_{ij}}{p-1}\right)I\left\{y_{ij}>0\right\}\\ \; & \;+\sum_{j=1}^{n_{i}}\left\{r_{yij}u_{yij}-\log\left[1+\exp\left(u_{yij}\right)\right]\right\}, \end{array}$$
  where $u_{yij}=\varphi_{y0}+\varphi_{y1}y_{ij}+\varphi_{y2}y_{i,j-1}$ or $u_{yij}=\varphi_{y0}+\varphi_{y1}y_{ij}+\varphi_{y2}x_{ij1}+\varphi_{y3}x_{ij2}+\cdots +\varphi_{y,k+1}x_{ijk}$($x_{ij1},x_{ij2},\ldots ,x_{ijk}$ are all missing covariables), $x_{i}=\left\{x_{i1},\ldots ,x_{in_{i}}\right\}$, $z_{i}=\left\{z_{i1},\ldots ,z_{in_{i}}\right\}$, $t_{i}=\left\{t_{i1},\ldots ,t_{in_{i}}\right\}$ and $u_{i}=\left\{u_{i1},\ldots ,u_{in_{i}}\right\}$.

(2) The logarithmic conditional distribution of $x_{mi}$ is
  $$\begin{array}{cc} \; & \log p(x_{mi}|x_{oi},u_{i},y_{i},z_{i},t_{i},b_{i},\Sigma ,\beta ,p,\phi ,\xi ,\alpha ,\varphi_{x})\\ \; & \propto \log p\left(y_{i},u_{i}|x_{i},z_{i},t_{i},b_{i},\theta \right)+\log p\left(r_{xi}|x_{i},\varphi_{x}\right)+\log p\left(x_{mi}|\alpha \right)\\ \; & =\log\prod_{j=1}^{n_{i}}p(y_{ij},u_{ij}|x_{ij},z_{ij},t_{ij},b_{i},\theta )+\log\prod_{k=1}^{q}p(r_{xijk}|x_{i},\varphi_{x})+\log p\left(x_{mi}|\alpha \right)\\ \; & =\sum_{j=1}^{n_{i}}\log p\left(y_{ij},u_{ij}|x_{ij},z_{ij},t_{ij},b_{i},\theta \right)+\sum_{k=1}^{q}\log p\left(r_{xijk}|x_{i},\varphi_{x}\right)+\log p\left(x_{mi}|\alpha \right)\\ \; & =-\frac{1}{\phi }\sum_{j=1}^{n_{i}}\frac{\mu_{ij}^{2-p}}{2-p}-\sum_{j=1}^{n_{i}}\left(\frac{y_{ij}}{\phi \left(p-1\right)\mu_{ij}^{p-1}}+\log\Gamma \left(u_{ij}\frac{2-p}{p-1}\right)+\log u_{ij}!+\log y_{ij}\right)I\left\{y_{ij}>0\right\}\\ \; & \;+\sum_{j=1}^{n_{i}}u_{ij}\left(\frac{2-p}{p-1}\log\frac{y_{ij}}{p-1}-\frac{\log\phi }{p-1}-\log\left(2-p\right)\right)I\left\{y_{ij}>0\right\}\\ \; & \;+\sum_{k=1}^{p}\log\left\{{\left(\frac{\exp(v_{ijk})}{1+\exp(v_{ijk})}\right)}^{r_{xijk}}{\left(1-\frac{\exp(v_{ijk})}{1+\exp(v_{ijk})}\right)}^{1-r_{xijk}}\right\}+\log p\left(x_{mi}|\alpha \right)\\ \; & \propto -\frac{1}{\phi }\sum_{j=1}^{n_{i}}\frac{\mu_{ij}^{2-p}}{2-p}-\sum_{j=1}^{n_{i}}\left(\frac{y_{ij}}{\phi \left(p-1\right)\mu_{ij}^{p-1}}\right)I\left\{y_{ij}>0\right\}\\ \; & \;+\sum_{k=1}^{p}\left\{r_{xijk}v_{ijk}-\log\left[1+\exp\left(v_{ijk}\right)\right]\right\}+\log p\left(x_{mi}|\alpha \right) \end{array},$$
  where $v_{xijk}=\varphi_{xk0}+\varphi_{xk1}x_{ij1}+\cdots +\varphi_{xkk}x_{ijk}+\varphi_{xk,k+1}r_{xij1}+\cdots +\varphi_{xk,2k-1}r_{xij,k-1}$ or $v_{xijk}=\varphi_{xk0}+\varphi_{xk1}x_{ij1}+\cdots +\varphi_{xkk}x_{ijk}+\varphi_{xk,k+1}y_{ij}$, $p(x_{mi}|\alpha )$ is specified by (12) and (13).
(3) The conditional distribution of $\varphi_{y}$ is
  $$\begin{array}{cc} \; & p\left(\varphi_{y}|Y,r_{y}\right)\propto p\left(r_{y}|Y,\varphi_{y}\right)p\left(\varphi_{y}\right)\\ \; & =\prod_{i=1}^{n}\prod_{j=1}^{n_{i}}p(r_{yij}|y_{i},\varphi_{y})p\left(\varphi_{y}\right)\\ \; & \propto \exp\left\{\sum_{i=1}^{n}\sum_{j=1}^{n_{i}}\left(r_{yij}u_{yij}-\log\left(1+\exp\left(u_{yij}\right)\right)\right)-\frac{1}{2}{\left(\varphi_{y}-\varphi_{y}^{0}\right)}^{T}B^{-1}\left(\varphi_{y}-\varphi_{y}^{0}\right)\right\}. \end{array},$$
  where $u_{yij}=\varphi_{y0}+\varphi_{y1}y_{ij}+\varphi_{y2}y_{i,j-1}$ or $u_{yij}=\varphi_{y0}+\varphi_{y1}y_{ij}+\varphi_{y2}x_{ij1}+\varphi_{y3}x_{ij2}+\cdots +\varphi_{y,k+1}$$x_{ijk}$($x_{ij1},x_{ij2},\ldots ,x_{ijk}$ are all missing covariables).
(4) The conditional distribution of $\varphi_{x}$ is
  $$\begin{array}{cc} \; & p(\varphi_{xk}|X,r_{x})\\ \; & \propto \exp\left\{\sum_{i=1}^{n}\sum_{j=1}^{n_{i}}\left(r_{xijk}v_{xijk}-\log\left(1+\exp\left(v_{xijk}\right)\right)\right)-\frac{1}{2}{\left(\varphi_{xk}-\varphi_{xk}^{0}\right)}^{T}C^{-1}\left(\varphi_{xk}-\varphi_{xk}^{0}\right)\right\} \end{array},$$
  where $v_{xijk}=\varphi_{xk0}+\varphi_{xk1}x_{ij1}+\cdots +\varphi_{xkk}x_{ijk}+\varphi_{xk,k+1}r_{xij1}+\cdots +\varphi_{xk,2k-1}r_{xij,k-1}$ or $v_{xijk}=\varphi_{xk0}+\varphi_{xk1}x_{ij1}+\cdots +\varphi_{xkk}x_{ijk}+\varphi_{xk,k+1}y_{ij}$.
(5) The conditional distribution of α is
  $$\begin{array}{cc} \; & p\left(\alpha |X,r_{x}\right)\propto p\left(X|\alpha ,r_{x}\right)p\left(\alpha \right)\\ \; & \propto \prod_{i=1}^{n}\prod_{j=1}^{n_{i}}p{\left(x_{ijq}|x_{ij1},\ldots ,x_{ij,q-1},\alpha_{q}\right)}^{r_{xijq}}\cdots p{\left(x_{ij2}|x_{ij1},\alpha_{2}\right)}^{r_{xij2}}\times p{\left(x_{ij1}|\alpha_{1}\right)}^{r_{xij1}}p\left(\alpha \right)\\ \; & =\prod_{i=1}^{n}\prod_{j=1}^{n_{i}}\prod_{k=1}^{q}p{\left(x_{ijk}|x_{ij1},\ldots ,x_{ij,k-1},\alpha_{q}\right)}^{r_{xijk}}p\left(\alpha \right)\\ \; & \propto \prod_{i=1}^{n}\prod_{j=1}^{n_{i}}\prod_{k=1}^{q}p{\left(x_{ijk}|x_{ij1},\ldots ,x_{ij,k-1},\alpha_{q}\right)}^{r_{xijk}}\exp\left\{-\frac{1}{2}{\left(\alpha -\alpha^{0}\right)}^{T}D^{-1}\left(\alpha -\alpha^{0}\right)\right\} \end{array}.$$
(6) According to (13), we know $x_{ij1}∼N\left(\mu_{ij1},\sigma_{x1}^{2}\right)$, and $p\left(\sigma_{x1}^{2}\right)∼IG\left(a_{x1},b_{x1}\right)$. Then the conditional distribution of $\sigma_{x1}^{2}$ is
  $$\begin{array}{cc} \; & p(\sigma_{x1}^{2}|x_{ij1},\alpha_{1})\\ \; & \propto {\left(\sigma_{x1}^{2}\right)}^{-a_{x1}-1}\exp\left(-\frac{b_{x1}}{\sigma_{x1}^{2}}\right)\prod_{i=1}^{n}\prod_{j=1}^{n_{i}}\frac{1}{\sqrt{2\pi }\sigma_{x1}^{2}}\exp\left\{-\frac{{\left(x_{ij1}-\mu_{ij1}\right)}^{2}}{2\sigma_{x1}^{2}}\right\}\\ \; & \propto {\left(\sigma_{x1}^{2}\right)}^{-a_{x1}-\frac{nn_{i}}{2}-1}\exp\left\{-\frac{1}{\sigma_{x1}^{2}}\left[b_{x1}+\frac{1}{2}\sum_{i=1}^{n}\sum_{j=1}^{n_{i}}{\left(x_{ij1}-\mu_{ij1}\right)}^{2}\right]\right\} \end{array}.$$
  Clearly, $p\left(\sigma_{x1}^{2}|x_{ij1},\alpha_{1}\right)∼IG\left(a_{x1}+\frac{nn_{i}}{2},b_{x1}+\frac{1}{2}\sum_{i=1}^{n}\sum_{j=1}^{n_{i}}{\left(x_{ij1}-\mu_{ij1}\right)}^{2}\right)$, where IG is the inverse gamma distribution. In addition, the conditional distributions of $\sigma_{x2}^{2},\sigma_{x3}^{2},\ldots ,\sigma_{xm}^{2}$ are the same as that of $\sigma_{x1}^{2}$.

The conditional distribution of the nonparametric part used in the Gibbs sampling algorithm is as follows.

(1) The logarithmic conditional distribution of ξ is
  $$\begin{array}{cc} \; & \log p(\xi |U,Y,X,T,\beta ,p,\phi ,b,\Sigma )\\ \; & \propto -\frac{1}{\phi }\sum_{i=1}^{n}\sum_{j=1}^{n_{i}}\frac{\mu_{ij}^{2-p}}{2-p}-\sum_{y_{ij}>0}\left(\frac{y_{ij}}{\phi \left(p-1\right)\mu_{ij}^{p-1}}\right)-\frac{1}{2\tau_{\xi }^{2}}\xi^{T}Q\xi \end{array}.$$
(2) The conditional distribution of $\tau_{\xi }^{2}$ is
  $$\begin{array}{cc} \; & p\left(\tau_{\xi }^{2}|\xi ,\delta \right)\propto {\left(\tau_{\xi }^{2}\right)}^{-\frac{rank(Q)}{2}-a_{\tau }-1}\exp\left\{-\frac{1}{2\tau_{\xi }^{2}}\xi^{T}Q\xi -\frac{b_{\tau }}{\tau_{\xi }^{2}}\right\} \end{array}.$$
  Clearly, $\tau_{\xi }^{2}|\xi ,\delta ∼IG\left(\frac{rank(Q)}{2}+a_{\tau },\frac{1}{2}\xi^{T}Q\xi +b_{\tau }\right)$, where IG is the inverse gamma distribution.
(3) The conditional distribution of $\delta_{\rho }$ is
  $$\begin{array}{c} p\left(\delta_{\rho }|\xi ,\tau_{\xi }^{2}\right)\propto {\left(\delta_{\rho }\right)}^{a_{\delta }-1/2}\exp\left\{-\delta_{\rho }\left(\frac{{\left(\xi_{\rho }-\xi_{\rho -1}\right)}^{2}}{2\tau_{\xi }^{2}}+b_{\delta }\right)\right\} \end{array}.$$
  Clearly, $p\left(\delta_{\rho }|\xi ,\tau_{\xi }^{2}\right)∼\Gamma \left(a_{\delta }+\frac{1}{2},\frac{{\left(\xi_{\rho }-\xi_{\rho -1}\right)}^{2}}{2\tau_{\xi }^{2}}+b_{\delta }\right)$, where Γ is the gamma distribution.

Finally, the conditional distributions of other parameters used in the Gibbs sampling are as follows.

(1) The logarithmic conditional distribution of β is
  $$\begin{array}{cc} \; & \log p(\beta |U,Y,X,T,\phi ,p,b,\Sigma ,\xi )\\ \; & \propto -\frac{1}{\phi }\sum_{i=1}^{n}\sum_{j=1}^{n_{i}}\frac{\mu_{ij}^{2-p}}{2-p}-\sum_{y_{ij}>0}\left(\frac{y_{ij}}{\phi \left(p-1\right)\mu_{ij}^{p-1}}\right)-\frac{1}{2}{\left(\beta -\beta^{0}\right)}^{T}A^{-1}\left(\beta -\beta^{0}\right) \end{array}.$$
(2) The logarithmic conditional distribution of *p* is
  $$\begin{array}{cc} \; & \log p(p|U,Y,X,T,\beta ,\phi ,b,\Sigma ,\xi )\\ \; & \propto -\frac{1}{\phi }\sum_{i=1}^{n}\sum_{j=1}^{n_{i}}\frac{\mu_{ij}^{2-p}}{2-p}-\sum_{y_{ij}>0}\left(\frac{y_{ij}}{\phi \left(p-1\right)\mu_{ij}^{p-1}}+\log\Gamma \left(n_{ij}\frac{2-p}{p-1}\right)\right)\\ \; & \;+\sum_{y_{ij}>0}n_{ij}\left(\frac{2-p}{p-1}\log\frac{y_{ij}}{p-1}-\frac{\log\phi }{p-1}-\log\left(2-p\right)\right)-\frac{{\left(\log\frac{p-1}{2-p}\right)}^{2}}{2\times 10,000} \end{array}.$$
(3) The logarithmic conditional distribution of ϕ is
  $$\begin{array}{cc} \; & \log p(\phi |U,Y,X,T,\beta ,p,b,\Sigma ,\xi )\\ \; & \propto -\frac{1}{\phi }\sum_{i=1}^{n}\sum_{j=1}^{n_{i}}\frac{\mu_{ij}^{2-p}}{2-p}-\sum_{y_{ij}>0}\frac{y_{ij}}{\phi \left(p-1\right)\mu_{ij}^{p-1}}+\sum_{y_{ij}>0}n_{ij}\left(-\frac{\log\phi }{p-1}\right)-\frac{{\left(\log\phi \right)}^{2}}{2\times 10,000} \end{array}.$$
(4) The logarithmic conditional distribution of b is
  $$\begin{array}{cc} \; & \log p(b|U,Y,X,T,\beta ,p,\phi ,\xi ,\Sigma )\\ \; & \propto -\frac{1}{\phi }\sum_{i=1}^{n}\sum_{j=1}^{n_{j}}\frac{\mu_{ij}^{2-p}}{2-p}-\sum_{y_{ij}>0}\left(\frac{y_{ij}}{\phi \left(p-1\right)\mu_{ij}^{p-1}}\right)-\sum_{i=1}^{n}\frac{1}{2}b_{i}^{T}\Sigma^{-1}b_{i} \end{array}.$$
  Thus, $\log p(b_{i}|U,Y,X,T,\beta ,p,\phi ,\xi ,\Sigma )$ is proportional to
  $$-\frac{1}{\phi }\sum_{j=1}^{n_{j}}\frac{\mu_{ij}^{2-p}}{2-p}-\sum_{j=1}^{n_{i}}\frac{y_{ij}}{\phi \left(p-1\right)\mu_{ij}^{p-1}}I\left\{y_{ij}>0\right\}-\frac{1}{2}b_{i}^{T}\Sigma^{-1}b_{i}.$$
(5) The conditional distribution of Σ is
  $$p\left(\Sigma |U,Y,X,T,\beta ,p,\phi ,b,\xi \right)\propto {\left|\Sigma \right|}^{\frac{-(\rho_{0}+n+d+1)}{2}}\exp\left\{-\frac{1}{2}tr\left[\Sigma^{-1}\left(\sum_{i=1}^{n}b_{i}b_{i}^{T}+R_{0}\right)\right]\right\}.$$
  Clearly, $\Sigma |U,Y,X,T,\beta ,p,\phi ,b,\xi ∼IW_{d}\left(n+\rho_{0},R_{0}+\sum_{i=1}^{n}b_{i}b_{i}^{T}\right)$, where $IW_{k}\left(\cdot ,\cdot \right)$ is the k-dimensional inverse Wishart distribution.
(6) The logarithmic conditional distribution of U is
  $$\begin{array}{c} \log p\left(U|Y,\phi ,p\right)\propto -\sum_{y_{ij}>0}\left(\log\Gamma \left(u_{ij}\frac{2-p}{p-1}\right)+\log u_{ij}!+\log y_{ij}\right)\\ \;\;\;\;\;+\sum_{y_{ij}>0}u_{ij}\left(\frac{2-p}{p-1}\log\frac{y_{ij}}{p-1}-\frac{\log\phi }{p-1}-\log\left(2-p\right)\right), \end{array}$$
  thus,
  $$\begin{array}{c} \log p\left(u_{ij}|Y,\phi ,p\right)\propto -\left(\log\Gamma \left(u_{ij}\frac{2-p}{p-1}\right)+\log u_{ij}!+\log y_{ij}\right)I\left\{y_{ij}>0\right\}\\ \;\;\;\;\;+u_{ij}\left(\frac{2-p}{p-1}\log\frac{y_{ij}}{p-1}-\frac{\log\phi }{p-1}-\log\left(2-p\right)\right)I\left\{y_{ij}>0\right\}. \end{array}$$

## Appendix A. Metropolis–Hastings Algorithm

To implement the Metropolis–Hastings algorithm, we assume that the current iteration values of $u_{ij}$, β, *p*, ϕ, $b_{i}$, and ξ are $u_{ij}^{\left(l\right)}$, $\beta^{\left(l\right)}$, $p^{\left(l\right)}$, $\phi^{\left(l\right)}$, $b_{i}^{\left(l\right)}$, and $\xi^{\left(l\right)}$, and the proposal distributions of the new random samples $u_{ij}^{*}$, $\beta^{*}$, $p^{*}$, $\phi^{*}$, $b_{i}^{*}$, and $\xi^{*}$ were selected as zero truncated Poisson distribution $f(u_{ij}^{\left(l\right)};\sigma_{n}^{2}\lambda |u_{ij}^{\left(l\right)}>0)$, multivariate normal distribution $N(\beta^{\left(l\right)},\sigma_{\beta }^{2}\Omega_{\beta })$, normal distribution $\log\frac{p-1}{2-p}∼N\left(p^{\left(l\right)},\sigma_{p}^{2}\right)$, normal distribution $\log\phi ∼N(\phi^{\left(l\right)},\sigma_{\phi }^{2})$, normal distribution $N(b_{i}^{\left(l\right)},\sigma_{b_{i}}^{2}\Omega_{b_{i}})$, and multivariate normal distribution $N(\xi^{\left(l\right)},\sigma_{\xi }^{2}\Omega_{\xi })$, respectively, where λ denotes the mean parameter of the Poisson distribution, *N* denotes the normal distribution, and $\sigma_{u}^{2}$, $\sigma_{\beta }^{2}$, $\sigma_{p}^{2}$, $\sigma_{\phi }^{2}$, $\sigma_{b_{i}}^{2}$, and $\sigma_{\xi }^{2}$ are the tuned parameters, respectively. Furthermore, $\Omega_{\beta }$, $\Omega_{b_{i}}$, and $\Omega_{\xi }$ are derived as

$$\begin{array}{c} \Omega_{\beta }={\left(\frac{2-p}{\phi }\sum_{i=1}^{n}\sum_{j=1}^{n_{i}}\mu_{ij}^{2-p}x_{ij}^{T}x_{ij}+\frac{p-1}{\phi }\sum_{y_{ij}>0}y_{ij}\mu_{ij}^{1-p}x_{ij}^{T}x_{ij}+A^{-1}\right)}^{-1},\\ \Omega_{b_{i}}={\left(\frac{2-p}{\phi }\sum_{j=1}^{n_{i}}\mu_{ij}^{2-p}\left.+\frac{p-1}{\phi }\sum_{j=1}^{n_{i}}y_{ij}\mu_{ij}^{1-p}I\left\{y_{ij}>0\right\}+\sum^{-1}\right)\right.}^{-1},\\ \Omega_{\xi }={\left(\frac{2-p}{\phi }\sum_{i=1}^{n}\sum_{j=1}^{n_{i}}\mu_{ij}^{2-p}B^{T}\left(t_{ij}\right)B\left(t_{ij}\right)+\frac{p-1}{\phi }\sum_{y_{ij}>0}y_{ij}\mu_{ij}^{1-p}B^{T}\left(t_{ij}\right)B\left(t_{ij}\right)+\frac{Q}{\tau_{\xi }^{2}}\right)}^{-1}, \end{array}$$

where $i=1,\ldots ,n,j=1,\ldots n_{i}$. Finally, we give the accepted probability of $u_{ij}$, β, *p*, ϕ, $b_{i}$, and ξ used in the Metropolis–Hastings algorithm as follows:

$$\begin{array}{c} \min\left\{\frac{p\left(u_{ij}^{*}|Y,p,\phi \right)f\left(u_{ij}^{\left(t\right)};\sigma_{u}^{2},\lambda |u_{ij}^{\left(l\right)}>0\right)}{p\left(u_{ij}^{\left(l\right)}|Y,p,\phi \right)f\left(u_{ij}^{*};\sigma_{u}^{2},\lambda |u_{ij}^{*}>0\right)},1\right\},\\ \min\left\{\frac{p(\beta^{*}|Y,X,Z,T,\phi ,p,b,\Sigma ,\xi )}{p(\beta^{\left(l\right)}|Y,X,Z,T,\phi ,p,b,\Sigma ,\xi )},1\right\},\\ \min\left\{\frac{p(p^{*}|U,Y,X,Z,T,\beta ,\phi ,b,\Sigma ,\xi )}{p(p^{\left(l\right)}|U,Y,X,Z,T,\beta ,\phi ,b,\Sigma ,\xi )},1\right\},\\ \min\left\{\frac{p(\phi^{*}|U,Y,X,Z,T,\beta ,p,b,\Sigma ,\xi )}{p(\phi^{\left(l\right)}|U,Y,X,Z,T,\beta ,p,b,\Sigma ,\xi )},1\right\},\\ \min\left\{\frac{p(b_{i}^{*}|U,Y,X,Z,T,\beta ,p,\phi ,\xi ,\Sigma )}{p(b_{i}^{\left(l\right)}|U,Y,X,Z,T,\beta ,p,\phi ,\xi ,\Sigma )},1\right\},\\ \min\left\{\frac{p(\xi^{*}|U,Y,X,Z,T,\beta ,p,\phi ,b,\Sigma )}{p(\xi^{\left(l\right)}|U,Y,Z,X,T,\beta ,p,\phi ,b,\Sigma )},1\right\} \end{array}.$$
